# Biomedical signals and machine learning in amyotrophic lateral sclerosis: a systematic review

**DOI:** 10.1186/s12938-021-00896-2

**Published:** 2021-06-15

**Authors:** Felipe Fernandes, Ingridy Barbalho, Daniele Barros, Ricardo Valentim, César Teixeira, Jorge Henriques, Paulo Gil, Mário Dourado Júnior

**Affiliations:** 1 Laboratory of Technological Innovation in Health (LAIS), Federal University of Rio Grande do Norte (UFRN), Natal, RN Brazil; 2 Department of Informatics Engineering, Univ Coimbra, CISUC-Center for Informatics and Systems of the University of Coimbra, Coimbra, Portugal

**Keywords:** Amyotrophic lateral sclerosis—ALS, Artificial intelligence, Biomedical signals, Chronic neurological conditions, Machine learning, Motor neuron disease, Signal processing

## Abstract

**Introduction:**

The use of machine learning (ML) techniques in healthcare encompasses an emerging concept that envisages vast contributions to the tackling of rare diseases. In this scenario, amyotrophic lateral sclerosis (ALS) involves complexities that are yet not demystified. In ALS, the biomedical signals present themselves as potential biomarkers that, when used in tandem with smart algorithms, can be useful to applications within the context of the disease.

**Methods:**

This Systematic Literature Review (SLR) consists of searching for and investigating primary studies that use ML techniques and biomedical signals related to ALS. Following the definition and execution of the SLR protocol, 18 articles met the inclusion, exclusion, and quality assessment criteria, and answered the SLR research questions.

**Discussions:**

Based on the results, we identified three classes of ML applications combined with biomedical signals in the context of ALS: diagnosis (72.22%), communication (22.22%), and survival prediction (5.56%).

**Conclusions:**

Distinct algorithmic models and biomedical signals have been reported and present promising approaches, regardless of their classes. In summary, this SLR provides an overview of the primary studies analyzed as well as directions for the construction and evolution of technology-based research within the scope of ALS.

## Background

Amyotrophic lateral sclerosis is a disease characterized by the progressive and irreversible degeneration of motor neurons, which causes deficits in the ability to control movement, breathing, and, in 50% of cases, in cognitive and behavioral functioning [1–3]. The cause of ALS is still unknown and there is no treatment to cure it. Hence, there are only alternatives of palliative care and medication to delay the progress of the disease [4, 5]. Diagnosing patients with ALS represents a challenging task due to its complex pathogenesis and the absence of specific biomarkers [6, 7]. The diagnosis is based on clinical presentation, progression of symptoms, and the exclusion of other diseases supported by tests such as Electromyography (EMG). Such a process requires an average of 10–18 months from the onset of symptoms to confirmation [8–11]. What is more, the diagnosis is considered slow and late given the characteristics of ALS, in which life expectancy after confirmation is of 2–5 years [12].

Despite being described by Jean-Martin Charcot more than 100 years ago [13], ALS is considered a rare disease, and, to this date, there are not many countries with records of epidemiological data. In a few European countries, as well as in the United States, epidemiological records show that the incidence rate of ALS is of 1–2 cases per 100,000 individuals per year, while the prevalence is approximately 5 cases per 100,000 individuals, which for van Es et al. [3] reflects the fast lethality of the disease. A worldwide increase in the number of ALS-affected individuals is expected, rising from 222,801 cases in 2015 to 376,674 by 2040, according to the projection made by Arthur et al. [14]. The aging of populations and the consequent rise in the number of individuals within the age group with a more considerable risk for ALS, which is of 60–79 years, represent the probable culprits for the 69% worldwide increase [14].

Considering the intrinsic aspects of ALS, it is critical to promptly search for diagnostic support systems, as well as for alternatives that intermediate essential communication, autonomy, and promote quality of life to patients. From this standpoint, several technology-based studies have been developed. These investigations typically provide auxiliary resources for diverse aspects regarding ALS, going from what pertains to patients and their caregivers to matters related to outpatient care in organizational health entities [15–17].

Technologies developed for health encompass and collaborate in positive progressions in remarkable ways, such as with the diagnosis of ALS [18, 19], monitoring of disease progression [20], monitoring of food intake [21], communication intermediation [22–25], autonomy [26], and other applications based in artificial intelligence, as it has been reviewed by Schwalbe and Wahl [27]. Automated systems for disease diagnosis, for instance, are computational tools composed of ML techniques that, based on the processing of biomedical signals, are capable of aiding the detection of neuromuscular disorders [28]. These systems contain expert information of specific domains, which provide health professionals with decision-making support and represent strategies and measures adopted in the care of patients [29].

Recently, in the context of ALS, Grollemund et al. [30] published a comprehensive review that presents and investigates ML models. Thus, it uses or combines different data types from individuals with ALS (clinical, genetic, biological clinical, and imaging), in three-class applications: diagnosis, prognosis, and risk stratification. In conclusion, the authors point to promising advances with this approach in the academic and clinical field in the ALS ecosystem. In this perspective, this SLR complements Grollemund et al. [30] in analyzing ML models in applications for ALS using specifically biomedical signals.

Biomedical signals consist of data from a studied physiological system and their processing aims mainly to extract relevant information [31, 32]. This information can enhance data-driven artificial intelligence techniques, especially ML algorithms, and it is used to support the diagnosis of various diseases [27]. There are several types of biomedical signals, as EMG, electroencephalogram (EEG), electrocardiogram (ECG), electrooculogram (EOG), gait rhythm (GR) and magnetic resonance imaging (MRI). Regarding the ML models, Artificial Neural Network (ANN), decision tree (DT), support vector machine (SVM), and K-Nearest Neighbor (KNN) are particular examples of techniques that have been extensively considered in the healthcare realm, including in the context of ALS [33–36].

## Objective

The chief goal of this systematic literature review is to investigate ML-based approaches, in tandem with the biomedical signals, that contribute to the practical and scientific advancement of aspects in the field of ALS. In this manner, it is expected to provide an overview of the matter at hand, considering the identification of the most-used biomedical signals and ML-based models, in addition to gathering details of primary studies, such as the purpose, the performance of algorithmic models, and experimental data, to identify strengths and opportunities for future researches.

## Methods

We have developed this research considering the systematic review guidelines proposed by Kitchenham [37]. In the perspective of investigating technological applications in ALS, this study aims at (i) identifying the most applied biomedical signals; (ii) identifying for what purposes those are used; and (iii) verifying the usage of ML techniques or intelligent approaches to the processing of those signals. Hence, the research questions (RQ) were elaborated on this premise (see Table 1, presented below).

**Table 1:** Research questions

RQ	Description
01	For what purpose is the processing of signals used?
02	What are the types of signals analyzed by the study?
03	What intelligent techniques are used in the study?
04	What is the performance of the analyzed techniques?
05	How many subjects are used to test or validate the study?

**Fig. 1:**
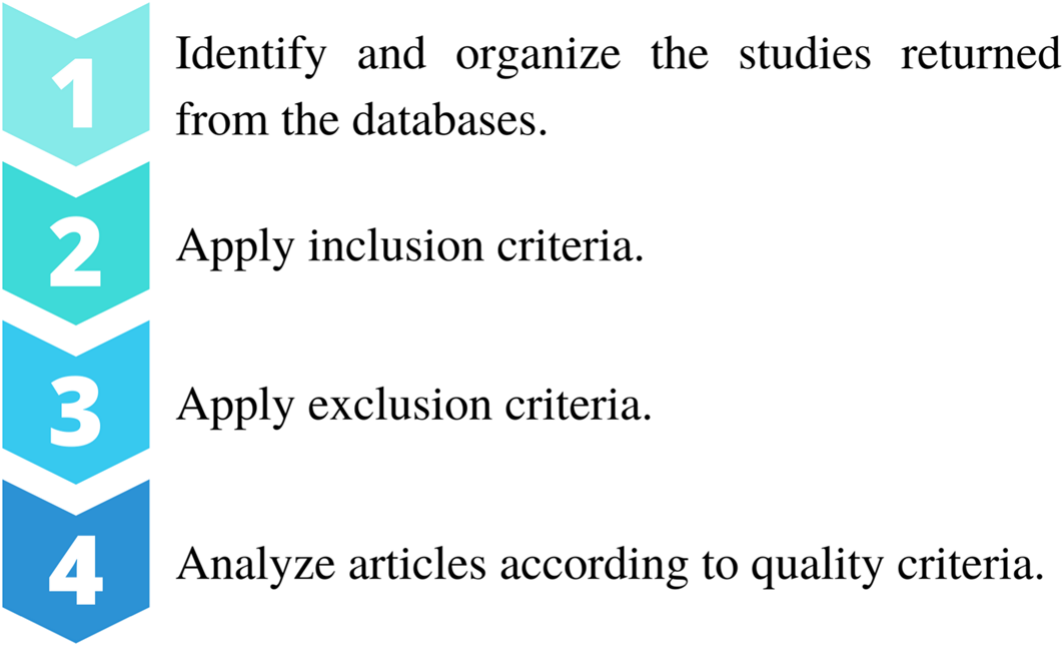
Methodology steps

The primary studies searching and screening process in the scientific databases were categorized into four stages, according to what is displayed in Fig. 1. In the first stage, an initial set of articles was selected from the output of searches carried out in the IEEE Xplore, Web of Science, Science Direct, Springer, and PubMed databases. The following search strings (STR) were used in this first stage:

- STR01: (((“signals processing” OR “signals biomedical”) OR (“smart systems” OR “machine learning” OR “artificial intelligence” OR “computational intelligence” OR “algorithm” OR “algorithms”)) AND (“amyotrophic lateral sclerosis” OR “als”));
- STR02: (((“signals processing” OR “signals biomedical”) OR (“intelligent systems” OR “machine learning” OR “artificial intelligence” OR “algorithms” OR “Computational Intelligence”)) AND (“amyotrophic lateral sclerosis” OR “als”)).

In the second stage, the predefined inclusion criteria (IC), presented in Table 2, were applied to the initial set of articles from the previous phase. Primarily, an IC delimits the boundaries or scope of the investigation and possibilities the generation of a new subset of papers with a more significant probability of answers to the RQ. In such a context, the subset includes research articles from the last ten years that have been published in journals and are directly related to the principal area of interest of this systematic review.

**Table 2:** Inclusion criteria

IC	Description
01	Articles published between 2009 and 2019
02	Research articles published in Journals
03	Articles in the areas of technology, engineering or computer science

In the third stage, after screening the articles through the IC, the verification and removal of duplicate papers were carried out. Besides, a filtering procedure—by considering title, abstract, and keywords—was performed to exclude papers that did not present specific terms related to the theme of this review. Such a process was guided by the exclusion criteria (EC) (see Table 3) and was executed through the Rayyan web application [38].

**Table 3:** Exclusion criteria

EC	Description
01	Duplicate articles
02	Studies not related to the processing of biomedical signals, ML, smart systems and data analysis of patients with ALS

In the fourth stage, the total reading of the filtered articles was performed. Hence, it was executed the quality assessment (QA) protocol (see criteria in Table 4). In the QA procedure, each criterion was attributed points measuring the relevance of the article to the target subject of this research. The points were distributed in the form of weights (*w*), considering suitable responses to the QA criteria, present in the primary studies, with 1.0 being the most relevant weight and 0 the lowest:

$$w_{\text {QA}} = \left\{ \begin{array}{ll} 1.0, & \text {yes},\; \text{fully}\; \text{describes} ,\\ 0.5, & \text {yes}, \; \text {partially} \; \text {describes},\\ 0, & \text {does}\;\text {not}\; \text {describe}.\\ \end{array} \right.$$

A score, the arithmetic mean of the points of the QA criteria (Eq. 1), was generated for each article. In this case, all articles that obtained a score greater than or equal to 0.5 ($0.5 \le \text{score} \le 1$) were selected for this research and constitute the final set of articles.

1 $$\text{score} = \frac{1}{\text {QA}}\sum _{i=1}^{\text {QA}} w_{\text {QA}_{i}}$$

Records relevant to each stage, as well as the data extracted from the articles, were properly gathered in spreadsheets and the Rayyan web application [38] for data extraction. Data, such as year of publication, authors, and possible responses to the RQ, were extracted from the set of articles of the fourth stage. They permitted the final analysis and fulfillment of the objectives of this systematic review.

**Table 4:** Quality assessment

QA	Description
01	Does the study clearly describe the types of biomedical signals?
02	Does the study describe how signal processing is performed (algorithmic techniques, intelligent systems)?
03	Does the study describe the process of the proposed application for ALS patients (does it detail how it was applied)?
04	Does the study clearly describe its scientific contributions to the evolution of ALS-related research?

## Results

**Fig. 2:**
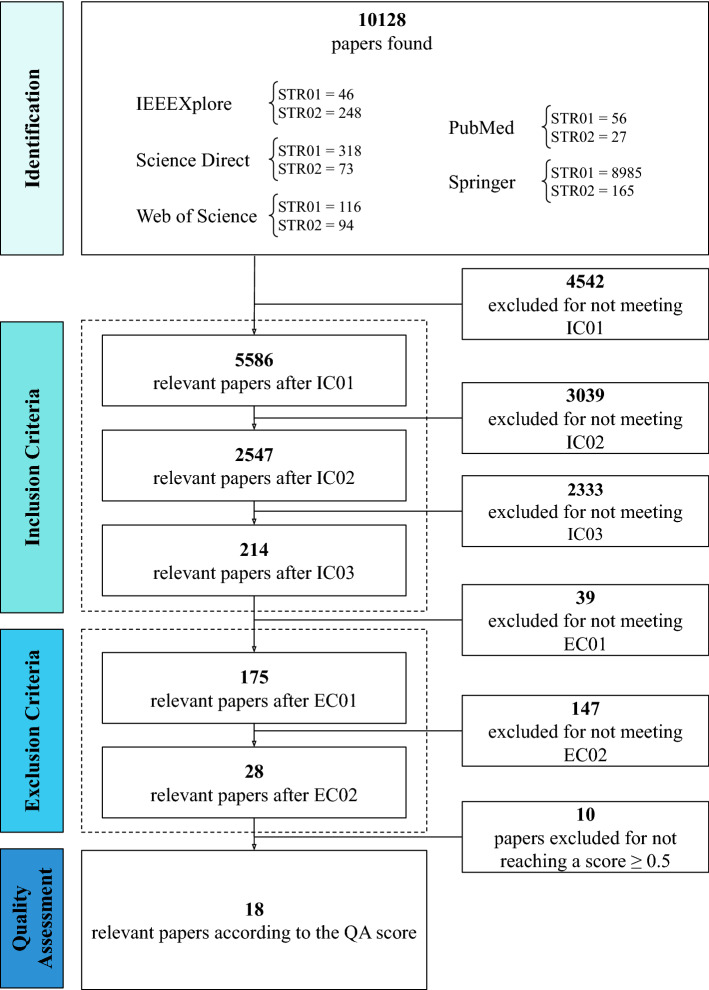
Result of the search and screening process of primary studies for this systematic review

The results obtained from the searching and screening process of primary studies are synthesized in Fig. 2. In the first stage, 10128 candidate articles were identified after searching with the STRs. In the subsequent phase, three refining procedures based on IC (Table 2) were applied, and 9914 papers were discarded for not meeting the IC. At this point, 214 articles were considered appropriate for inclusion and analysis in the following stage. In stage three, the applied filters, based on the EC (Table 3), removed 186 articles amongst duplicates and those missing the target terms of the search. In this manner, 28 studies were selected for full-text reading and assessment through the QA criteria. After the QA procedure, the fourth and latter phase, 18 papers exceeded the pre-established minimum score, according to the result presented in the respective column in Table 5, and were included for analysis and definitive investigation in this review.

**Table 5:** Set of selected articles and their main characteristics

Study	Year	Score	Goal	Signals	Dataset	Subjects	Best model	Performance (%)		
						HC/ALS/OD		Acc	Spe	Sen
Chatterjee et al. [39]	2019	1.0	Diagnosis	EMG	Public	–/8/7	SVM	98.58	99.5	97.66
Zhang et al. [40]	2014	1.0	Diagnosis	EMG	Local	11/10/–	LDA	–	100	90
Hazarika et al. [41]^a^	2019	1.0	Diagnosis	EMG	Public	10/8/7 and 4/4/4	QDC and QDC	99.03 and 100	99.58 and 100	96 and 100
Gokgoz and Subasi [42]	2014	1.0	Diagnosis	EMG	Local	10/8/7	SVM	92.55	90.3	96.33
Ambikapathy et al. [43]	2018	0.875	Diagnosis	EMG	Public	–	ANN	96.6	100	93.7
Doulah et al. [44]	2014	1.0	Diagnosis	EMG	Public	10/8/7	KNN	98.8	100	98
Vallejo et al. [45]	2018	1.0	Diagnosis	EMG	Public	10/8/7	ANN	98	97.5	100
Gokgoz and Subasi [46]	2015	1.0	Diagnosis	EMG	Public	10/8/7	RF	96.67	94.75	99.58
Xia et al. [47]	2015	0.875	Diagnosis	GR	Public	16/13/35	SVM	96.55	94	100
Ren et al. [48]	2017	1.0	Diagnosis	GR	Public	16/13/35	MLP	–	–	–
Khorasani et al. [49]	2016	0.875	Diagnosis	GR	Public	16/13/–	FHMM	93.1	93.75	92.31
Welsh et al. [50]	2013	1.0	Diagnosis	MRI	Local	31/32/–	SVM	71.5	–	–
Ferraro et al. [51]	2017	0.75	Diagnosis	MRI	Local	78/123/64	RF	91	92	91
Miao et al. [55]	2020	1.0	Communication	EEG	Local	–/18/–	BLDA	90	–	–
Liu et al. [53]	2017	0.875	Communication	EEG	Local	–/5/–	KNN and LDA	95.25	–	–
Sorbello et al. [52]	2018	0.75	Communication	EEG	Local	4/4/–	LDA	–	–	–
Mainsah et al. [54]	2015	0.875	Communication	EEG	Local	–/10/–	DSLM	76.39	–	–
van der Burgh et al. [56]	2017	1.0	Survival	MRI	Local	–/135/–	DLN	84.4	–	–

*HC* healthy controls, *OD* other diseases, *Acc* accuracy, *Spe* specificity, *Sen* sensitivity, *EMG* electromyography, *EEG* electroencephalogram, *MRI* magnetic resonance imaging, *GR* gait rhythm, *SVM* support vector machine, *RF* random forest, *LDA* linear discriminant analysis, *QDC* quadratic classifier, *ANN* Artificial Neural Network, *KNN* k-Nearest Neighbor, *MLP* Multilayer Perceptron, *FHMM* Factorial hidden Markov model, *BLDA* Bayesian linear discriminant analysis, *DSLM* dynamic stopping with language model, *DLN* Deep Learning Networks

^a^The study did experiments on two datasets

**Fig. 3:**
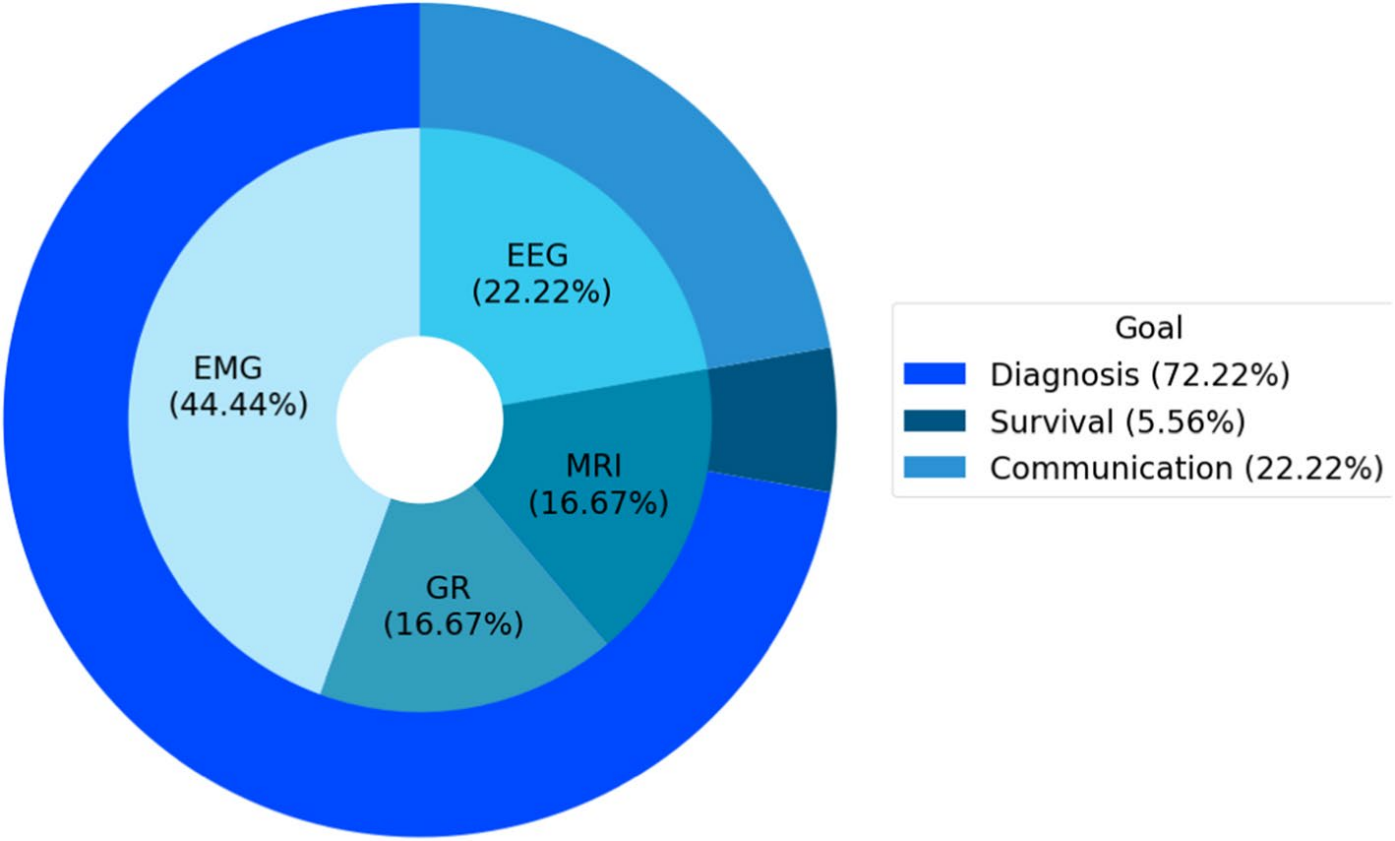
Summary of the signals used and their objectives

In sum, considering the 18 articles included in this research, the results presented in Fig. 3 evidence three major classes of probable practical applications of biomedical signals processing and machine learning within the context of the ALS disease: diagnosis (or classification), communication, and survival prediction. In addition to categorizing the purposes of such studies, Fig. 3 highlights the number of biomedical signals used and the respective classes that utilized them. Four distinct types of signals were identified: EMG, EEG, GR, and MRI.

Of the analyzed studies, 44.44% focus on the processing of the EMG signal, the most used biomedical signal (see Fig. 3), and specifically for classification. That is, for the diagnosis of individuals amongst healthy controls (HC), ALS patients, and, in some cases, other diseases (OD). With the same objective, especially for classification, 16.67% of the studies use GR and 11.11% MRI. The MRI signal was additionally used in a particular article for survival prediction of ALS-afflicted individuals, which represents 5.56%. In the communication class, 22.22% of the studies focus exclusively on the approach through the processing of the EEG signal, being this the only one presented for that purpose. This first general analysis of the studies, identifying the purposes of the articles and the signals used, answers research questions RQ01 and RQ02.

Other significant and specific characteristics extracted from the 18 articles included in this study are summarized in Table 5, to support the analysis and answer research questions RQ03, RQ04, and RQ05. Regardless of the classes observed, diagnosis, communication, or survival prediction, all studies used ML algorithms. Alternative algorithmic models were employed and, according to the performance analysis of the algorithm concerning accuracy (Acc), specificity (Spe), or sensitivity (Sen) metrics of evaluation, the best or the only proposed model of each work is shown in Table 5, as well as their respective performances.

**Fig. 4:**
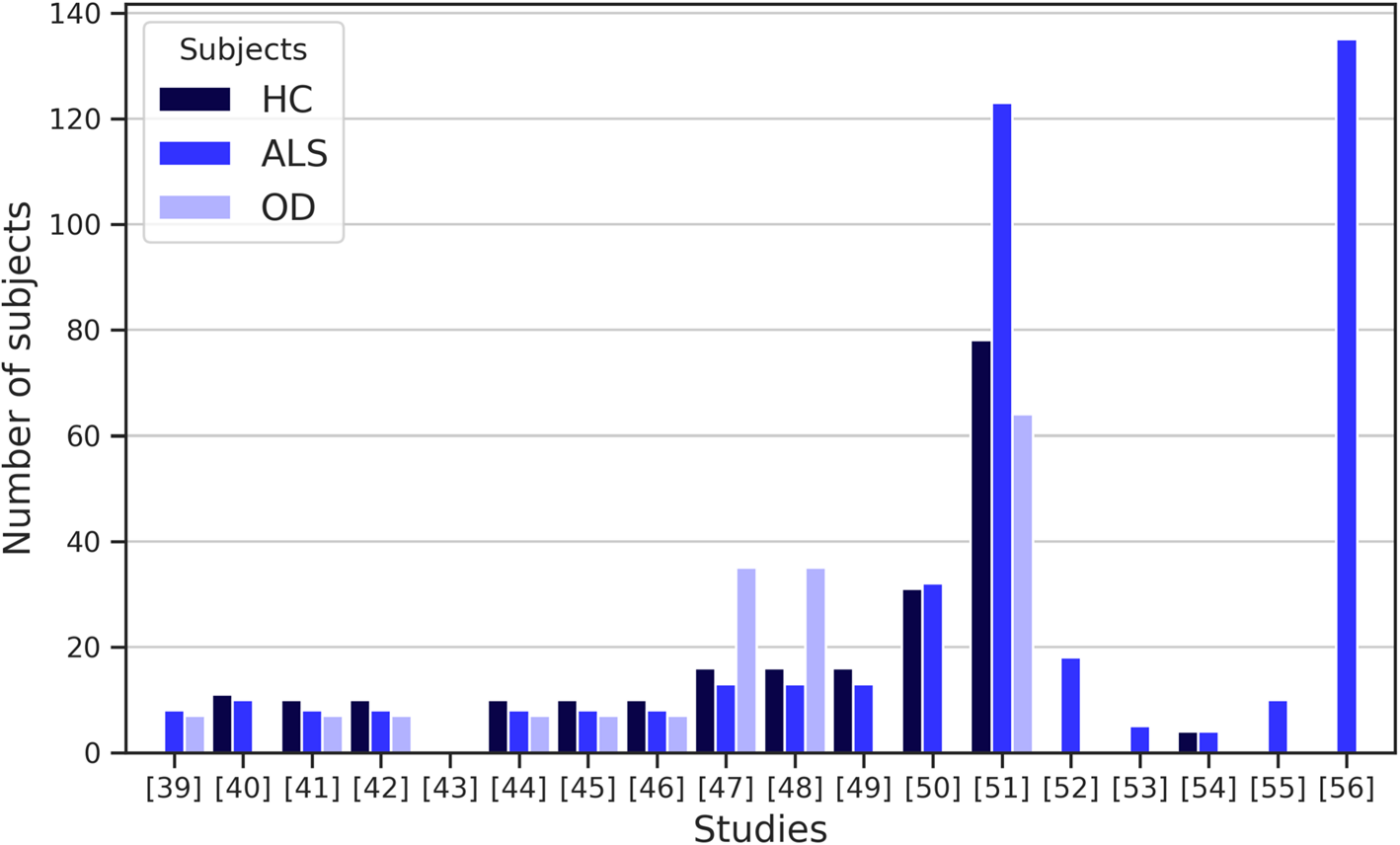
Number of individuals used in the studies

For testing, validating, and appraising the proposed approaches in the studies, the algorithmic techniques were applied to a set of data from individuals, distributed in different group combinations of HC, ALS, and/or myopathy, or other neurological diseases. The number of individuals and the type of participating groups in the experiments of each study is specified in Table 5 and summarized in Fig. 4. Moreover, Table 5 describes the source of the dataset and specifies whether they come from public or local repositories.

### Description of the diagnosis studies

**Fig. 5:**
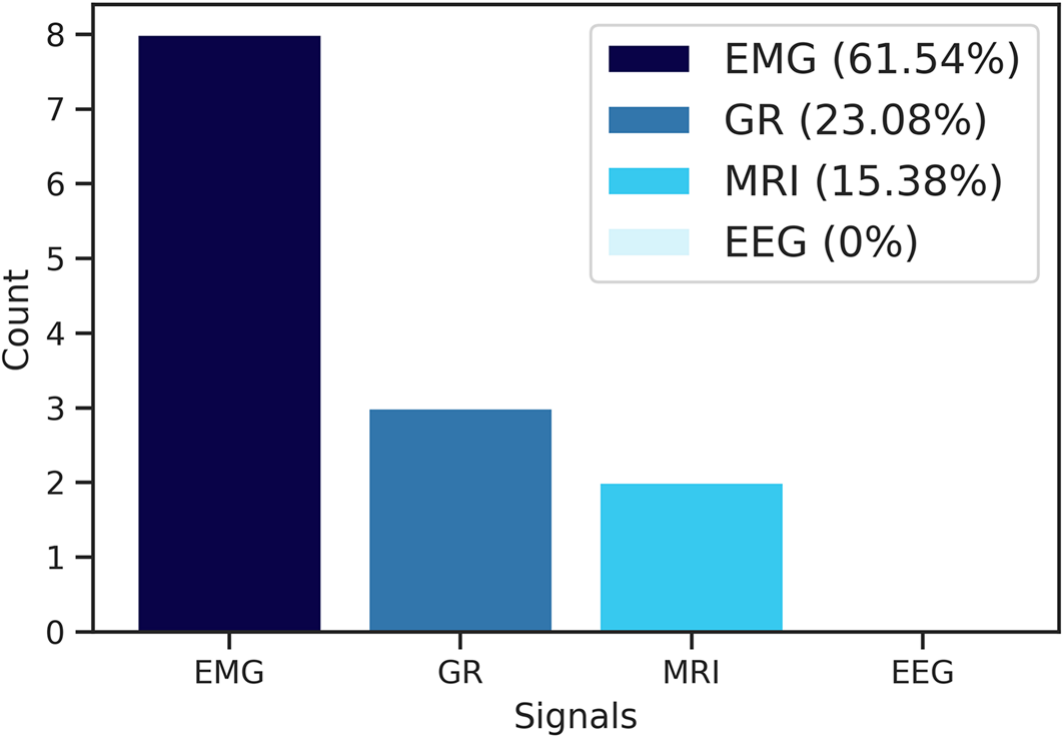
Number of studies by type of biomedical signal defined in the diagnosis class

Diagnosis of ALS patients is the most numerous task described among the selected papers, accounting for 72.22% of the studies. Considering only this class, Fig. 5 presents an overview of the number of biomedical signals employed. The use of the EMG signal stands out, being addressed in 61.54% of the studies aimed at diagnosis [39–46]. GR is applied in 23.08% of the studies [47–49] and MRI in 15.38% [50, 51]. The EEG signal is unused for this purpose.

In the studies, the focus on the development of strategies to reduce the noise of collected biomedical signals is evident, intending to discover the most significant features to enhance the performance of the algorithmic models, therefore reflecting on the accurate classification of individuals. In the case of the EMG signal, Gokgoz and Subasi [42] show the performance gain in the classification of HC, ALS, and OD with different ML algorithms, such as KNN, ANN, and SVM, after applying the multiscale principal component analysis (MSPCA) noise removal technique combined with the multiple signal classification (MUSIC) feature extraction technique.

In this study, the most satisfactory performance was of the SVM model, with 92.55% Acc. In the following year, also addressing the EMG signal, Gokgoz and Subasi [46] proposed another study focusing on noise reduction and selection of features. Hence, in this research, the authors developed a structure to eliminate noise, also employing the MSPCA technique, with a novel strategy based on Discrete Wavelet Transform (DWT) for feature extraction. The authors performed experimental tests, with and without the noise-removing structure and feature extraction, utilizing three different DT algorithmic models: CART, C4.5, and random forest (RF). Similarly to their previous study, Gokgoz and Subasi [46] achieved satisfactory results in their second work in the classification of HC, ALS, and OD, and demonstrated that the use of the MSPCA noise removal method in conjunction with feature extraction using DWT improved the performance of the RF algorithmic model, which obtained in the best case 96.67% Acc using the EMG signal. In both studies, three distinct groups of subjects were considered: HC $= 10$, ALS $= 8$ and OD $= 7$.

Vallejo et al. [45] adopted the DWT approach to decompose EMG signals and generate the hyperspace of features that aims at selecting the most relevant features through the fuzzy entropy technique to feed an ML algorithm based on an ANN. To verify and validate the proposed method, the authors used a feedforward ANN with four layers of seven neurons each, except for the output layer, and the log-sigmoid activation function. The ANN, acting with the DWT and fuzzy entropy approaches, obtained 98% Acc in the best result. Such a fact indicated that the proposed strategies for feature selection and extraction improved the classification process of individuals into three distinct groups, including HC, ALS, and/or myopathy. In the experiments, the authors included ten healthy subjects, eight with ALS and seven with OD.

In Doulah et al. [44], the DWT approach was applied to decompose, filter, and extract relevant features from preprocessed EMG signals. Preprocessing encompassed two methods proposed for the classification of subjects in HC and ALS, and/or myopathy. In the direct EMG method with DWT, features are extracted after a frame-by-frame sequential analysis. For the second approach, a set of features was selected through the procedure of dominant motor unit action potential (MUAP), and, based on the DWT decomposition, the key features for classification were extracted. Doulah et al. [44] verified both proposed methods in a KNN classifier. The authors evaluated the performance of the algorithm by operating distinct settings on three separate sets of data corresponding to HC, ALS, and OD subjects (10, 8 and 7 subjects, respectively). Both processes presented significant results for the classification of two and three sets (HC, ALS, and OD). The Dominant-MUAP method may be highlighted for its consistent performance, reaching out 98.8% Acc using three sets.

Chatterjee et al. [39] presented a new generalization of the Stockwell transform (ST), called Modified Window ST (MWST), for preprocessing the EMG signals to generate a more representative feature matrix (or a time–frequency plane). MWST parameters $\alpha$, $\beta$, $\gamma$, and $\delta$ which affect the shape of the window or the energy concentration in the time–frequency plane, are defined in an optimized way through the Particle Swarm Optimization (PSO) metaheuristic algorithm. After applying the MWST technique to the EMG signals of eight individuals with ALS and seven with myopathy, four features are extracted from the matrix to serve as input to four ML models: SVM, KNN, Naïve Bayes, and DT. The approach proposed by Chatterjee et al. [39] presented significantly better results in the classification of individuals between ALS and myopathy when compared to the conventional ST method. The SVM model achieved the most satisfactory performance with 98.28% Acc.

Four distinct methods of feature extraction from EMG signals in the time–frequency domain have been addressed ST, Synchro-extracting Transform, Wigner Ville distribution, and Short-Time Fourier Transform. The four approaches applied to EMG signals generated images and, using the Gray Level Co-occurrence Matrix technique, 20 features were extracted with each method, and a set of 80 features was originated. To optimize the performance of the ML model proposed by the authors, an ANN with a hidden layer of 10 neurons and tan sigmoid activation function combined with a subset containing the most suitable combination of 15 features (from a finite set of possibilities) was defined through a genetic algorithm (GA), implemented with the KNN classifier to carry out fitness evaluation of the different combinations of the GA population. The strategy proposed by Ambikapathy et al. [43] to aid diagnosis using ANN obtained promising and statistically significant results in the process of classifying individuals between HC and ALS (86.6% Acc, 86.6% Spe, 86.6% Sen), HC, ALS or myopathy (82.2% Acc, 81.89% Sen, 91.31% Spe), and ALS or myopathy (96.6% Acc, 93.7% Sen, 100% Spe).

Zhang et al. [40] developed a method to characterize patterns in surface EMG signals based on three markers/features for supporting the diagnostic: clustering index, kurtosis of the EMG signal amplitude histogram, and kurtosis of EMG zero crossing-rate expansion. Furthermore, the linear discriminant analysis (LDA) ML algorithm was applied to the process of discriminating subjects as HC and ALS, obtaining as input data a feature vector originated from the concatenation of vectors of the mentioned features. The experiments carried out in this study relied on the analysis of data from 10 subjects with ALS and 11 from HC. Each of the three features displayed unique promising results. When used synergistically in an approach that combines them with the LDA algorithm, it rendered a more robust technique that presents even more significant outcomes, presenting a Spe of 100% and Sen of 90%. Such an aspect indicates favorable perspectives to be used as a diagnosis support application.

Hazarika et al. [41] proposed a novel process assessment and inference system (PAIS) with a robust structure for preprocessing and extracting features from EMG signals. This structure is composed of procedures that initially evaluate the EMG signal features via an approach involving partitioning strategies of the input data (called multi-view direct) and decomposition by DWT, followed by the application of multidomain multiview discriminant correlation analysis (mmDCA). mmDCA analyzes the correlation of features, verifies redundancy, eliminates irrelevant features, and synchronizes them from partitions in a single vector. The mmDCA produced vector is incorporated into different ML models, such as KNN, and fed to a feedforward back-propagation neural network (FFBP-ANN) with two hidden layers of 8 neurons each, a conventional density-based linear classifier (LDC), a quadratic classifier (QDC) and SVM.

Each algorithm performance was evaluated positively in the classification of subjects between HC, ALS, and myopathy classes in two real-time EMG signal datasets. In the first dataset, containing ten healthy subjects, eight with ALS and seven with OD, and in the second dataset, containing four healthy subjects, four with ALS and four with OD, the QDC model presented the most robust results: 99.03% Acc and 100% Acc, respectively.

The studies mentioned thus far show the potential and the importance of processing the EMG biomedical signal to provide consistent and elementary features or data to the learning of intelligent algorithms and, consequently, to help with the diagnosis of ALS. However, as an alternative to EMG signals for diagnosis, the studies [47–49] investigated the performance of ML models with strategies that process data from GR biomedical signals.

A study by Xia et al. [47] carried out feature extraction from GR signals in five time series records, based on statistical analysis. Features such as mean value, standard deviation, maximum and minimum value, skewness, and kurtosis were computed and defined. The authors also proposed the extraction of three more features using the Lempel–Ziv complexity, fuzzy entropy, and Teager–Kaiser energy operator statistical methods. Furthermore, Xia et al. [47] executed an approach of selecting a subset of features based on three procedures, with the first being a statistical analysis of the features, followed by a performance evaluation of classification algorithms and ultimately the application of the hill-climbing optimization algorithm to find out and define the optimal subset of features.

After selection, a series of experiments with ML algorithms was performed considering the optimal subset as the input data for four classifiers: SVM, RF, a feedforward ANN with sigmoid activation function, and KNN. The dataset used for the experiments included data from 16 healthy subjects, 13 with ALS and 35 with OD. All classifiers showed good performance in the binary classification between HC and ALS, and HC and OD (neurodegenerative) in addition to ALS. The most satisfactory performance was obtained by the SVM technique, with 96.55%.

Ren et al. [48] performed a strategy to extract and select features from five time series of GR signals. For this, the researchers proposed an approach that utilizes the empirical mode decomposition (EMD) method to extract features from the partitioning of the GR signal time series, therefore producing six components—five of which are used and one discarded—that were promptly submitted to statistical analysis using Kendall’s Coefficient of Concordance method. With this, the purpose is to measure the significance and relationship of the features. Next, a calculation that employed the amplitudes of the components was made through the procedure of Ratio for Energy Change. Such a process comprehends to a dimensionality reduction technique based on principal component analysis (PCA) is applied to define the final set of features.

The strategy proposed by the authors was evaluated in five classifiers: Naïve Bayes, SVM, RF, Multilayer Perceptron (MLP) and Simple Logistic Regression. Unlike the performance evaluation of the ML models considered in Table 5, in this study the area under the ROC curve (AUC) is used for performance assessment. The most significant results for classifying subjects between HC and ALS (16 and 13 subjects, respectively) were presented by the feedforward MLP model, with an AUC value of 0.934. The average value of the AUC considering the five classifiers was 0.898 and, in general, the approach indicates promising results.

Differently from other researches developed for diagnostic, in the study of Khorasani et al. [49], a generalization of the Hidden Markov Model (HMM) and the classification model named the factorial hidden Markov model (FHMM), the recognition of patterns and the classification of subjects in HC or ALS from GR time series is proposed. After preprocessing the signal to remove outliers, the GR signal is segmented into fifty time series. And some features, such as mean or variance, are extracted to feed the FHMM. The method was assessed with data from 16 subjects with HC and 13 with ALS. Further, its performance was compared to the traditional HMM model and the Least-Squares SVM (LS-SVM) algorithm with Gaussian kernels. With an Acc value of 93.1%, the FHMM classification model displayed superior performance in HC or ALS recognition.

Scarcely explored in the context of diagnosing patients with ALS, strategies based on neuroimaging and ML are still challenging. In 2013, Welsh et al. [50] proposed an approach when implementing the SVM algorithm to classify subjects in HC or ALS through the analysis of data from functional magnetic resonance imaging (fMRI). The fMRI time series data were preprocessed and underwent a set of robust procedures for the extraction and selection of features, including the consecutive execution of strategies, such as PCA and Independent Components Analysis (ICA), as well as the creation of maps (or vectors) of correlation coefficients from different brain regions. After that, the data were provided to the linear kernel SVM algorithm, and its performance was evaluated. For the experiments, data from 32 patients diagnosed with ALS and 31 people from HC were provided. Welsh et al. [50] indicated the values were modest (71.5% Acc) in the classification of diseases as ALS using fMRI at rest.

A few years after the fMRI study by Welsh et al. [50], Ferraro et al. [51] developed a method for classifying individuals with motor neuron diseases, including ALS, based on the multimodal structural MRI with an ML algorithm. The MRI data were divided into distinct regions of interest and underwent analysis using literature and statistics software. The evaluation of the proposed diagnosis approach was performed in an RF model. The performance of the algorithm in classifying individuals in HC and ALS, specifically with the combined MRI model, was expressive. For the experiments, data from 78 individuals from HC, 123 subjects with ALS, and 64 subjects with OD were used. The model developed showed an Acc of 91% concerning the classification of subjects with ALS and subjects from HC. The results of both studies [50, 51] typically indicate that only data from MRI-based strategies are insufficient to obtain good performance in the classification. Studies with neuroimaging and ML for ALS are yet restricted. Nevertheless, the results are promising for the development of a system capable of assisting in ALS diagnosing.

### Communication improving studies

As a result of the progressive degeneration of the upper and/or lower motor neurons in the brainstem region, people diagnosed with ALS lose the ability to speak and interact with the environment. Technological resources developed for communication are crucial to ensure the well-being of those patients. About 22% of the studies included in this review [52–55], have developed artifacts that promote the communication improvement class of this SLR, exclusively through the EEG biosignal.

In a study conducted by Sorbello et al. [52], a framework was proposed through the operation of a brain–computer interface (BCI) system to control a humanoid robot and promote minimal autonomy to patients with ALS. Generally, the system structure, called brain–computer robotic interface (BCRI), is composed of a BCI system, EEG and eye-tracking devices, and a network system to connect the BCI system to the robotic system. The ML LDA algorithm is used after preprocessing and EEG feature extraction to correctly classify and translate the user action into control commands for the humanoid robot. The authors evaluated the proposal by conducting experiments on four subjects at the HC and four subjects with ALS. The results were satisfactory, and the proposed framework for enabling communication for patients with ALS was validated after all participants were able to control the humanoid robot.

Liu et al. [53] developed an approach by applying the concepts of fractal dimension (FD) and Fisher’s criterion to optimize the selection of EEG channels and the characterization of the data obtained from the signal. In this manner, the authors aimed at improving the classification capacity of an ML algorithm in a BCI system for patients with ALS. Two methods for estimating FD, Grassberger-Procaccia (GPFD) and Higuchi (HFD), were implemented. The key features of 30 EEG channels were extracted and concatenated into a single vector to serve two algorithmic models: KNN and LDA. After tests performed on five subjects with ALS, the results were satisfactory and the GPFD method surpassed the HFD. The performances of the two algorithms, KNN and LDA, were significant and similar, with 95.25% Acc, when compared with the input data containing the 30 EEG channels.

The existence of a simple interface with an accurate and fast information transfer rate is essential to maintain communication efficiency in a BCI system based on EEG signals for people with ALS. For addressing such matter, Mainsah et al. [54] developed a data-driven Bayesian early stopping algorithm, called DS, to optimize the feature selection process of an ERP-based P300 BCI speller, in which ERP stands for event-related potentials. Besides, a variation of the DS is proposed with the application of statistical modeling through Bayesian inference for language predictability, called DSLM. Features correlated with the user’s interest were extracted from the EEG signal to train the stepwise LDA classifier. In the research, the designated online tests were performed with 10 subjects with ALS. Both DS and DSLM algorithms proved to be efficient in minimizing the character selection time and with an average accuracy of 75.40% and 76.39%, respectively. There was no statistical difference between the algorithms.

In the same context of Mainsah et al. [54] and Miao et al. [55] proposed an ERP-based BCI display approach using as strategy a new speller paradigm with peripherally distributed stimuli with the possibility of feedback in the center of the display. The EEG signals were recorded and analyzed using preexisting software from the BCI platform. The features were extracted from data acquired offline from 16 electrodes to train the Bayesian LDA (BLDA) classifier and subsequently utilization of the trained model in an online system test. The proposed method was evaluated concerning the conventional matrix speller paradigm. The experiments were carried out on 18 subjects with ALS. Even obtaining an Acc of 90% in its most efficient performance, the results presented by the BLDA algorithm do not reveal any significant difference between the proposed approach and the conventional approach. However, patients with ALS were able to operate the system effectively.

### Survival prediction studies

The survival prediction of patients with ALS is empirically defined based on, generally, the analysis of clinical data. Only one of the studies included in this review is dedicated to such a prediction. In the study developed by van der Burgh et al. [56], a model for predicting survival (short, medium, or long) of patients with ALS is proposed by combining clinical data, neuroimaging, and a robust ANN-based ML technique named Deep Learning Networks (DLN). Four scenarios were defined for the application of the DLN algorithm. Finally, the first situation was based only on clinical data. The second and third scenarios utilized MRI images and included structural connectivity and brain morphology data. The latter situation included a combination of the previous three. To each of those, a model was implemented. Furthermore, the performance of algorithms was evaluated through a database that contained data of 135 subjects with ALS. The model that combined clinical and MRI data revealed superior performance (84.4% of Acc value) and was presented as a viable strategy for predicting the survival of patients with ALS. The remaining models displayed intermediate results, although they indicated promising approaches.

## Discussions

**Fig. 6:**
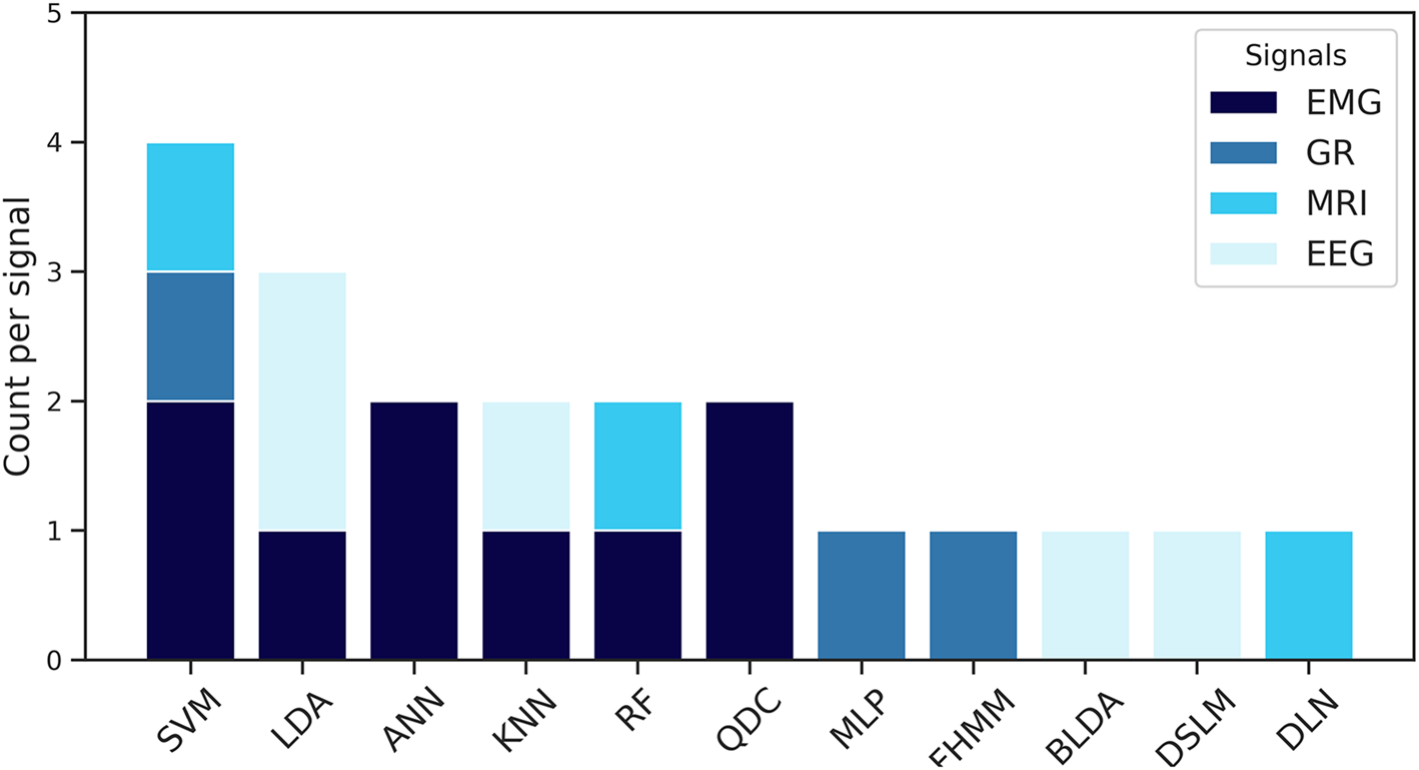
Quantitative of the best algorithmic models and the respective biomedical signals

This systematic literature review explored approaches based on computational intelligence. Besides, to process biomedical signals considering the scope of ALS, it functioned in a synergistic and complementary manner. A set of 18 articles was included and reviewed, and three major classes of applications were found: aid to diagnostic, communication enabling, and survival prediction. The most adequate algorithmic models and the respective biomedical signals responsible for providing data were identified and quantified (see Fig. 6).

**Fig. 7:**
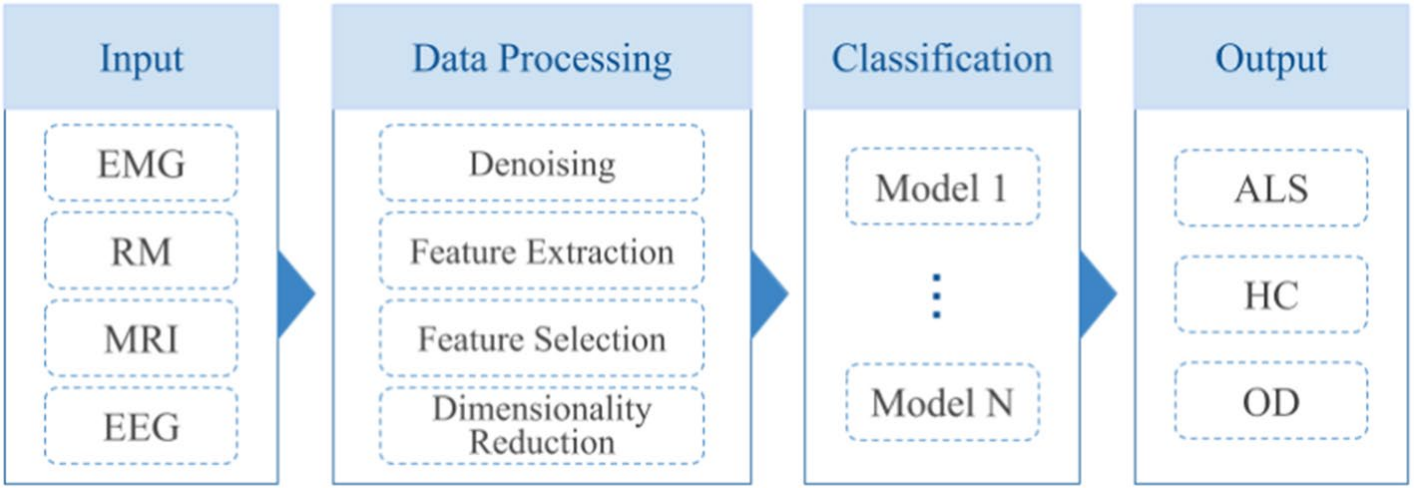
Generic pipeline: generalized scheme for solving classification problems

Based on the analysis of the 13 articles that addressed the support to the diagnosis of patients with ALS, regardless of the biomedical signal or ML algorithm used, it is possible to define a standard methodological scheme (a pipeline) general to all studies, which is broadly depicted in Fig. 7. Except for Khorasani et al. [49], who investigated a new classification algorithm, the studies suggest approaches or methods for the data treatment process that may enhance the training stage and, consequently, the classification stage. This data treatment process, which includes the feature extraction and selection phases, for instance, is important to eliminate noise, redundancy, and reduce the data dimensionality, in addition to maximizing the performances of the algorithms through the provision of refined and consistent data [57]. The various ML models implemented were presented as techniques for evaluating and validating the proposals of the studies. However, they were elementary techniques in the diagnosis process that are present in all articles.

The studies [39, 41–48, 51] about diagnosis support, in addition to ALS, also tested approaches for binary or multi-label classification considering other neurological conditions, such as myopathy, Parkinson’s disease, Huntington’s disease, predominantly upper motor neuron disease, and ALS-mimic disorders.

Regarding the four articles belonging to the communication class, two distinct categories can be observed in the analyzed studies. The first one is that of the study conducted by Sorbello et al. [52], which aims at complementing and adapting a BCI system with a humanoid robot to provide not only communication but also a minimum of autonomy. In the second identified category, the studies [53–55] suggest alternative approaches that include ML to optimize character selection time in a BCI system. These approaches range from the optimization of EEG electrodes to intelligent customization of the interface. The importance of BCI systems in promoting communication is evident. These systems are widely utilized in research to establish a communication pathway between the human brain and external devices, recognizing voluntary changes in the brain activity of their users [58–63].

Despite the research focused on the development of BCI systems, there are limitations regarding their home use. One of the primary reasons why BCI have not been introduced into the domestic environment is the character selection time. Specifically, the time still is considered slow and inaccurate when it comes to approaches that do not use brain signals, and also the need for electrodes connected to the head of the patient [47, 54, 64]. Other approaches to human–computer interaction systems that do not necessarily involve brain signals by EEG can be seen in Pinheiro et al. [65], Hori et al. [66], Fathi et al. [67], Harezlak et al. [68], Villanueva et al. [69], Królak e Strumiłło [24], Zhao et al. [70], Liu et al. [71] and Aharonson et al. [22].

The only survival prediction study with ALS patients analyzes how challenging it is to develop systems for such a purpose. The study [56] indicates that MRI and the DLN technique are promising for survival prediction and suggests a more significant exploration of the field of neuroimaging. Also, the research reveals the importance and benefits of patients’ clinical data in the process of predicting survival at the three levels of ALS. This observation, in combination with the analysis made thus far, reveals both the absence and the possibility of using clinical data for diagnosis. Correlated with the survival aspect, recent studies indicate it is possible to apply ML approaches with digital biomarkers using the speech signal to monitor the progression of ALS [72], including applications for automatic classification of the ALS Functional Rating Scale (ALSFRS) [73, 74].

Regarding ML algorithms, it is observed that they are specifically supervised in all studies. The type of biomedical signal varies only in the diagnosis studies, with EMG being the most used signal, followed by GR and MRI. The EEG signal is applied solely for communication enabling applications. The MRI-based biomedical signal is used both in diagnosis and survival prediction applications. Schuster et al. [75] affirm that MRI-based biomarkers are currently seldom used for aiding the identification of ALS. This observation is complemented by the results presented in this SLR, which also reports the limited number of neuroimaging-based studies aimed at diagnosis support applications and survival prediction of the ALS disease, despite the potential mentioned by van der Burgh et al. [56]. In addition to these biomedical signs mentioned so far, studies show the feasibility of using the speech biosignal for the early diagnosis of ALS, as indicated by Wang et al. [76], Suhas et al. [77], An et al. [78], Vieira et al. [79], and Wisler et al. [80], and tracking changes in individuals with bulbar ALS [81].

The 18 studies carried out experimental tests with datasets of healthy subjects and subjects with ALS or other neurological diseases. 50% of the studies used local or proprietary datasets. The other 50% of the investigations collected data from public online repositories. In some cases, like those for diagnosis and communication, except in the study carried out by Ferraro et al. [51], the limitation in the number of patients with ALS is evident (see Fig. 4). These results suggest that it is still challenging to develop and validate a robust study with a more considerable number of subjects with ALS or in an outpatient setting.

## Conclusion

This article introduces an SLR protocol to investigate relevant studies from the last ten years (2009–2019) that address ML techniques and biomedical signal processing. It may contribute to the advancement of research within the context of ALS. Based on 18 primary studies, the results exhibit strategies to minimize problems and/or promote means for diagnosis support, communication, and survival prediction. Considering the analyzed studies, 88.89% of those report the importance of treating biomedical signals for providing robust and consistent data for ML algorithmic models.

Furthermore, it can be observed that there is a predominance in the type of biomedical signals used by studies in the categories of communication and prediction of survival, being exclusively and respectively the EEG signals and MRI images. For the diagnosis class, in particular, three types of raw data are reported, namely EMG (61.54%), GR (23.08%), and MRI (15.38%). Regarding ML algorithmic models and analyzing the most satisfactory performances, SVM is the most used, followed by LDA and ANN techniques. Even though the 18 articles selected use ML, except for one study that proposed a new algorithm. In general, limited to the objectives of this SLR, the literature suggests and dedicates itself to the treatment of biomedical signals.

The studies are promising, but there are, nonetheless, significant aspects to be explored. When it comes to the diagnosis, the studies may be applied in outpatient clinics for practical assistance, in cases that have yet been unconfirmed of ALS, or even so in the early stages of the disease. Moreover, the use of big data approaches with patient’s clinical data might contribute to the conclusive results and remains open for investigations. That includes the field of survival prediction. Concerning the approaches for communication improvement, there are unanswered questions about the use of BCI in the domestic environment, considering its aspects, costs, as well as efficient interfaces that prevent fatigue, discomfort, and optimization of the electrodes for EEG signals acquisition.

## Data Availability

Not applicable.

## Abbreviations

Acc: Accuracy
ALS: Amyotrophic lateral sclerosis
ALSFRS: ALS Functional Rating Scale
ANN: Artificial Neural Network
AUC: Receiver operating characteristic curves
BCI: Brain computer interface
BCRI: Brain computer robotic interface
BLDA: Bayesian linear discriminant analysis
DLN: Deep Learning Network
DS: Dynamic stopping
DSLM: Dynamic stopping with language model
DT: Decision tree
DWT: Discrete Wavelet Transform
EC: Exclusion criteria
ECG: Electrocardiogram
EEG: Electroencephalogram
EMD: Empirical mode decomposition
EMG: Electromyography
EOG: Electrooculogram
FD: Fractal dimension
FHMM: Factorial hidden Markov model
fMRI: Functional magnetic resonance imaging
GA: Genetic algorithm
GPFD: Grassberger-Procaccia fractal dimension
GR: Gait rhythm
HC: Healthy controls
HFD: Higuchi fractal dimension
HMM: Hidden Markov model
IC: Inclusion criteria
ICA: Independent components analysis
KNN: K-Nearest Neighbor
LDA: Linear discriminant analysis
LDC: Density-based linear classifier
LS-SVM: Least-squares support vector machine
ML: Machine learning
MLP: Multilayer perceptron
mmDCA: Multidomain multiview discriminant correlation analysis
MRI: Magnetic resonance imaging
MSPCA: Multiscale principal component analysis
MUAP: Motor unit action potential
MUSIC: Multiple signal classification
MwST: Modified window Stockwell transform
OD: Other diseases
PAIS: Process assessment and inference system
PCA: Principal component analysis
PSO: Particle Swarm Optimization
QA: Quality assessment
QDC: Quadratic classifier
RF: Random forest
RQ: Research questions
Sen: Sensitivity
SLR: Systematic literature review
Spe: Specificity
ST: Stockwell transform
STR: Search strings
SVM: Support vector machine and (*w*): weights
